# Pulmonary 64-MDCT angiography with 50 mL of iodinated contrast material in an unselected patient population: a feasible protocol*

**DOI:** 10.1590/0100-3984.2014.0115

**Published:** 2016

**Authors:** Henrique Simão Trad, Gustavo Santos Boasquevisque, Tiago Rangon Giacometti, Catherine Yang Trad, Orlando Salomão Zoghbi Neto, Clovis Simão Trad

**Affiliations:** 1 MD, Radiologist at the Central de Diagnóstico Ribeirão Preto (Cedirp), Ribeirão Preto, SP, Brazil.; 2 Medical Physicist at the Central de Diagnóstico Ribeirão Preto (Cedirp), Ribeirão Preto, SP, Brazil.

**Keywords:** Tomography, X-ray computed, Contrast media, Angiography, Pulmonary embolism

## Abstract

**Objective:**

To propose a protocol for pulmonary angiography using 64-slice multidetector computed tomography (64-MDCT) with 50 mL of iodinated contrast material, in an unselected patient population, as well as to evaluate vascular enhancement and image quality.

**Materials and Methods:**

We evaluated 29 patients (22-86 years of age). The body mass index ranged from 19.0 kg/m^2^ to 41.8 kg/m^2^. Patients underwent pulmonary CT angiography in a 64-MDCT scanner, receiving 50 mL of iodinated contrast material via venous access at a rate of 4.5 mL/s. Bolus tracking was applied in the superior vena cava. Two experienced radiologists assessed image quality and vascular enhancement.

**Results:**

The mean density was 382 Hounsfield units (HU) for the pulmonary trunk; 379 and 377 HU for the right and left main pulmonary arteries, respectively; and 346 and 364 HU for the right and left inferior pulmonary arteries, respectively. In all patients, subsegmental arteries were analyzed. There were streak artifacts from contrast material in the superior vena cava in all patients. However, those artifacts did not impair the image analysis.

**Conclusion:**

Our findings suggest that pulmonary angiography using 64-MDCT with 50 mL of iodinated contrast can produce high quality images in unselected patient populations.

## Abstract (pt)

**Objetivo:**

Propusemos um protocolo com 50 mL de contraste, sem seleção de população e testamos o contraste vascular e a qualidade de imagem.

**Materiais e Métodos:**

Vinte e nove pacientes com idades entre 22 e 86 anos, com índice de massa corporal de 19,0 a 41,8 kg/m^2^, realizaram angiografia pulmonar em equipamento de 64 detectores. Foram injetados 50 mL de contraste iodado em acesso venoso periférico, na taxa de 4,5 mL/s^2^, com técnica de *bolus tracking* na veia cava superior. Dois radiologistas experientes avaliaram o realce vascular e a qualidade de imagem.

**Resultados:**

A densidade média no tronco pulmonar, ramos direito e esquerdo, artérias pulmonares inferiores direita e esquerda foram, respectivamente, 382, 379, 377, 346 e 364 unidades Hounsfield. Artérias subsegmentares foram avaliadas em todos os casos. Artefatos por restos de contraste na veia cava superior estavam presentes em todos os casos, sem prejudicar a avaliação das imagens.

**Conclusão:**

Angiotomografia pulmonar pode ser realizada com 50 mL de contraste iodado, sem necessidade de seleção dos pacientes e apesar de suas características físicas, com boa qualidade de imagem.

## INTRODUCTION

Pulmonary computed tomography angiography (PCTA) has long been in use and has become the gold standard for the evaluation of patients with suspected pulmonary embolism^(1,2)^. In the beginning, when single-detector scans were used, it was difficult to detect peripheral emboli^(3)^ and long scan times demanded large volumes of contrast material^(4,5)^.

Despite advances in multidetector computed tomography (MDCT) and increases in the speed of acquisition of large volumes of data^(6,7)^, there has been no significant reduction in the volume of iodinated contrast material required for pulmonary angiography in routine practice. In fact, there is extensive data in the literature demonstrating that PCTA is more efficacious and better able to depict small vessels than are other diagnostic methods^(8)^. Although authors have reported reducing the contrast volume to 30-40 mL^(9,10)^, their studies were conducted in selected populations under special conditions.

Following a more pragmatic approach, as discussed extensively in a recent study conducted by Hartmann et al.^(7)^, we proposed a simple standard protocol with 50 mL of iodinated contrast material, in an unselected patient population, in which we tested vascular enhancement and image quality.

## MATERIALS AND METHODS

Our study sample included 32 patients-25 females and 7 males, with a mean age of 53 years (range, 22-90 years)- suspected of having acute or chronic pulmonary thromboembolism, who were referred to our outpatient clinic between September 2012 and December 2013. The body mass index ranged from 19.0 kg/m^2^ to 41.8 kg/m^2^ (mean, 28.1 kg/m^2^).

All patients underwent PCTA in a 64-slice MDCT (64-MDCT) scanner (LightSpeed VCT; GE Healthcare, Milwaukee, WI, USA), with acquisition parameters of 1.25 mm digital collimation, 0.5 s tube rotation, pitch ratio of 1375:1, tube voltage of 100 kV, and automatic tube current modulation (SmartmA; GE Healthcare). Because of technical constraints, a tube voltage of 120 kV was used in three patients: one who was obese (135 kg); and two who could not raise their arms over their heads.

**Figure 1:**
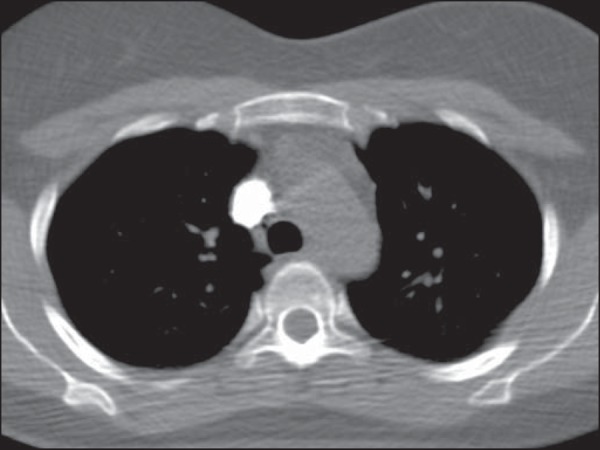
Bolus tracking control image with a fully filled superior vena cava.

We injected 50 mL of iodinated contrast material (iopamidol 612 mg/mL, 300 mg I/mL, Iopamiron 300; Bracco, Milan, Italy) into an antecubital venous access, at a preferred rate of 4.5 mL/s. In four patients, the rate of injection was reduced to 4.0 mL/s due to poor venous access. The total injection time ranged from 11.0 s (at 4.5 mL/s) to 12.5 s (at 4.0 mL/s). In all patients, the injection of contrast material was followed by the infusion of 30 mL of normal saline at the same injection rate. Bolus tracking was applied in the superior vena cava at the aortic arch level (Figure 1), with no established threshold or automatic trigger for acquisition. Alternatively, acquisition was triggered when the superior vena cava was seen to be completely filled with contrast material. The scan acquisition delay was 6-7 s, sufficient time for the complete intravascular buildup of material in the pulmonary arterial tree.

**Figure 2:**
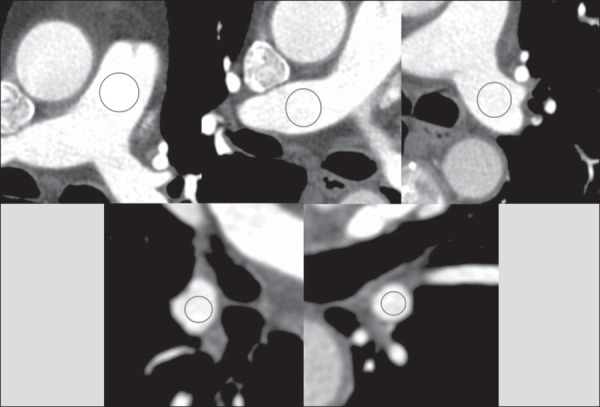
Assessment of vascular enhancement in the pulmonary trunk; right and left main pulmonary arteries; and right and left inferior pulmonary arteries.

Two experienced radiologists assessed image quality, in terms of the capacity to identify thromboembolism and signs of pulmonary hypertension, as well as enhancement of subsegmental pulmonary arteries and the presence of superior vena cava streak artifacts. As can be seen in Figure 2, vascular enhancement was assessed in the pulmonary trunk; in the right and left main pulmonary arteries; and in the right and left inferior pulmonary arteries. For each image, the regions of interest (ROIs) encompassed most of the vascular lumen. The signal-to-noise ratio (SNR) and contrast-to-noise ratio (CNR) were calculated for every ROI, noise being defined as the standard deviation of each. The SNR was calculated by dividing the ROI signal by its noise. In addition, in the pectoral muscles and the deep paraspinal muscles, at least four ROIs were measured bilaterally and averaged, the result being considered representative of the muscle signal. The CNR was calculated by subtracting the vascular signal from the muscle signal and dividing the result by the noise.

## RESULTS

In our study sample, the total scan time ranged from 2.2 to 2.8 s, with an mean of 2.5 s.

The overall mean arterial density, in Hounsfield units (HU), was 384 HU (range, 189-634 HU). Specifically, the mean ± standard deviation was 395 ± 99 HU for the pulmonary trunk; 391 ± 94 HU and 390 ± 91 HU for the right and left main pulmonary arteries, respectively; and 315 ± 96 HU and 378 ± 91 HU for the right and left inferior pulmonary arteries, respectively. The overall mean SNR and CNR were 28.7 and 25.2, respectively, for the right main pulmonary arteries; 19.0 and 16.6, respectively, for the left main pulmonary arteries; 23.1 and 20.2, respectively, for the right inferior pulmonary arteries; and 26.1 and 22.7, respectively, for the left inferior pulmonary arteries. Subsegmental arteries were analyzed in all patients.

In 8 patients, we identified pulmonary thromboembolism, which involved major branches in 3 and segmental to subsegmental arteries in 5. Signs of pulmonary hypertension were seen in 5 patients, all of whom showed enlarged pulmonary arteries, clear hypertrophy of the right ventricle being observed in only one case. However, among the patients with signs of pulmonary hypertension, only 2 showed thromboembolism in the current PCTA.

Streak artifacts from residual contrast material in the superior vena cava were present in all patients, although it did not impede image analysis of the pulmonary arterial tree in any cases. The mean dose length product was 362.1 mGy* cm, and the mean effective equivalent dose was 5.08 mSv.

## DISCUSSION

It is almost common knowledge that the duration of the contrast injection, or contrast column, should be approximately equal to the sum of the scan time and scan delay, as has previously been described^(7)^. In addition, it has been demonstrated that a large iodine load is not warranted at a scan time less than 5 s, because that is much shorter than the duration of the contrast bolus^(9)^. In the present study, the acquisition parameters applied yielded a mean scan time of 2.5 s, with a mean delay of 6.6 s and total scan times just above 9 s. That is quite well suited to an 11-s contrast column.

Reducing the volume of contrast material employed has several advantages, and the current recommendation is to use the smallest volume and lowest dose of iodine that will yield a diagnostic result^(11)^. Despite the controversy in the literature about the risk and incidence of contrast-induced kidney injury and its outcome^(12,13)^, some authors have identified a relationship between kidney injury and the volume of contrast material used^(14)^. In addition, from a financial point of view, reducing the volume of contrast material used translates to a reduction in health care system costs.

A major point of discussion in the literature is which contrast material and iodine concentration achieve the best results. Initially, contrast material with a higher concentration of iodine was shown to produce better results^(15)^. However, several authors^(16-18)^ have reported that, at same iodine delivery rate (IDR)-defined as iodine concentration multiplied by injection rate^(19)^-there is no statistical difference among different iodine concentrations in terms of the contrast enhancement of the pulmonary arterial tree. In a recent study involving a porcine model, Behrendt et al.^(20)^ showed that, at the same IDR, the greatest intravascular enhancement was achieved with moderate iodine concentrations. In a previous study, Behrendt et al.^(19)^ had shown that, for chest imaging, better contrast enhancement was achieved with 300 mg I/mL than with 370 mg I/mL, at the same IDR. In the present study, we employed an IDR of 1.35 g I/s, with a total iodine dose of 15 g. In all patients, we achieved a arterial density superior than the minimum attenuations of blood required to see all acute and chronic pulmonary venous thromboemboli (93 and 211 HU, respectively), as calculated by Wittram^(21)^. Because the major quality issue in CT angiography is intravascular enhancement and not tissue enhancement, the total dose of contrast is not an important factor. The major factor to consider is the acquisition time (scan time plus delay time) in relation to the contrast bolus. The studies mentioned above have shown that a major index to consider is the IDR, rather than the contrast iodine concentration or total iodine dose. In a study conducted by Wu et al.^(10)^, a total iodine dose of 9.6 g was injected in patients receiving a low dose (30 mL) of contrast material, with no reduction in image quality, and the authors concluded that the volume of contrast can and should be significantly reduced in carefully selected groups of patients. In the present study, no patient selection would be made on any clinical basis. Therefore, we chose to use 50 mL of contrast at a 4.5 mL/s injection rate, given that, as previously discussed, this combination would prove to be the most suitable in terms of the technical aspects. In addition, with no prior clinical selection, we figured that some problems related to cardiovascular conditions, such as cardiac insufficiency, could cause a loss of image quality at lower contrast volumes. We did not encounter the inspiration artifact discussed in the literature^(7,22)^, which could render a nondiagnostic result, in any of the cases evaluated here.

In accordance with recent data^(7,9,23,24)^, we used low kilovoltage acquisition in our patients, with only three exceptions in which, due to clinical conditions, we used a tube voltage of 120 kV in order to avoid nondiagnostic results. We maintained the mean radiation dose at the suggested levels^(7)^.

The major limitation of our study is the absence of a control group for the comparison of image quality, which limits the statistical analysis. Because the idea was to assess the image quality obtained with the proposed protocol alone, we did not work with a comparative control population. In fact, the main idea was to show that good image quality can be achieved at a lower contrast volume, taking into consideration only the technical aspects discussed earlier, without patient selection. Other limitations of our study are related to the small size of the patient population, as well as to its origin. Because ours is an outpatient clinic, we have not worked with patient populations that are clinically quite complicated.

## CONCLUSIONS

It seems that PCTA can be performed in a 64-MDCT scanner with 50 mL of iodinated contrast material in unselected patient populations, regardless of patient characteristics, and still produce images of high quality. Further clinical studies, involving larger patient samples in outpatient, emergency, and hospital settings, are needed in order to validate this protocol.
